# Head Vessel Reintervention Post-Type A Dissection Repair With a Hybrid Stent Prosthesis

**DOI:** 10.1016/j.jaccas.2025.104944

**Published:** 2025-10-29

**Authors:** Ryaan EL-Andari, Jeremy L. Rempel, Michael C. Moon

**Affiliations:** a Division of Cardiac Surgery, University of Alberta, Edmonton, Alberta, Canada; b Department of Radiology and Diagnostic Imaging, University of Alberta, Edmonton, Alberta, Canada

**Keywords:** AMDS Hybrid Prosthesis, aortic dissection, endovascular intervention

## Abstract

**Background:**

Adverse aortic remodeling may occur after the repair of acute type A aortic dissection (ATAAD), with branch vessel involvement being implicated due to retrograde perfusion of the false lumen (FL).

**Case Summary:**

A 61-year-old man presented with adverse aortic remodeling following ATAAD repair with the AMDS Hybrid Prosthesis, with maximal growth in zone 2 of 9 mm over 1 year. Tears were identified in the right common carotid artery and innominate artery, which were treated with endovascular stenting. There was no further growth in zones 0 to 3 three years postprocedure.

**Discussion:**

This case demonstrates cessation in the progression of aortic degeneration following head vessel endovascular treatment. In aortic degeneration following ATAAD repair, branch vessel involvement should be considered for potential contributions to adverse remodeling.

**Take-Home Message:**

Branch vessel involvement and endovascular intervention should be considered in cases of adverse aortic remodeling following ATAAD repair, potentially reducing the need for open reintervention.

## Graphical Abstract

**Figure:**
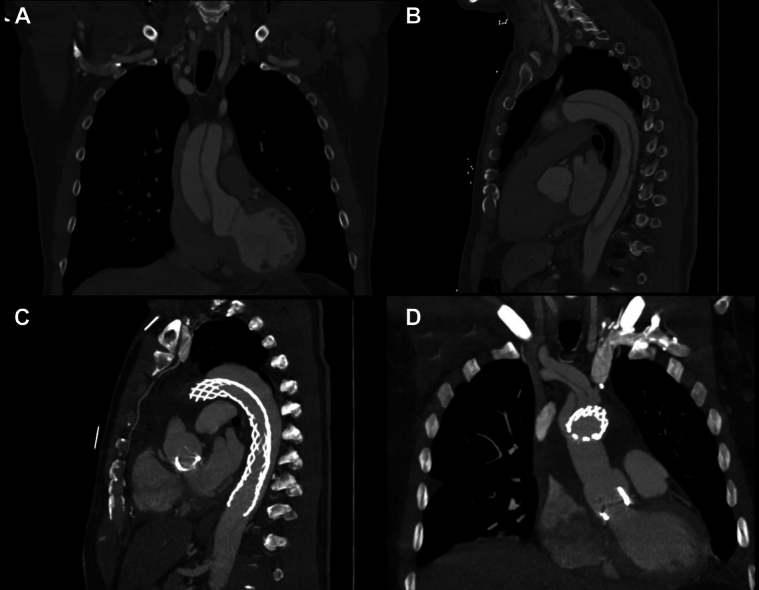

Acute type A aortic dissection (ATAAD) is a surgical emergency and carries a high risk of mortality.^1^ In recent years, the management of ATAAD has advanced, and short-term outcomes have improved. Consequently, there has been a shift in focus toward aortic remodeling and reintervention in cases of adverse aortic remodeling.^2^, ^3^, ^4^ The AMDS Hybrid Prosthesis (Artivion) is a hybrid arch device used to treat DeBakey I aortic dissection, and reinterventions in patients who have received the AMDS are infrequent.^2^^,^^5^

Importantly, the role of branch vessel involvement in aortic remodeling following dissection repair is not well understood. While there has been increasing research in this field suggesting the impact of branch vessel involvement on aortic remodeling, there is limited direct evidence showing a causal relationship between branch vessel disease and adverse aortic remodeling.^2^^,^^6^ Herein, we report the case of a 61-year-old patient with aneurysmal degeneration of the aortic arch following ATAAD repair, with the AMDS successfully treated with stenting of the head vessels.

## Case Report

The patient provided written informed consent to publish their case.

## History of Presentation

A 61-year-old man initially presented to the hospital with chest pain and differential upper extremity blood pressures.

## Past Medical History

The patient had a past medical history including hypertension, obstructive sleep apnea, and diverticulosis.

## Differential Diagnosis

Given the patient's presentation with chest pain and differential blood pressures, ATAAD was the most likely diagnosis with the differential including myocardial infarction, pulmonary embolism, and tension pneumothorax.

## Investigations

**Figure 1:**
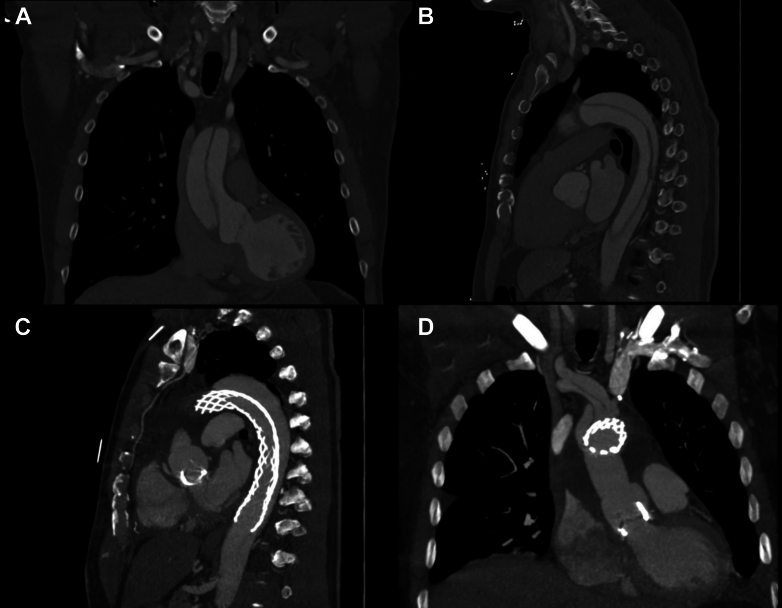
Preoperative Computed Tomography Scans Preoperative computed tomography (CT) demonstrating an acute type A aortic dissection (A and B). Postoperative CT following ascending aortic repair and AMDS Hybrid Prosthesis implantation (C and D).

The patient underwent a computed tomography, which revealed an ATAAD with a maximal diameter of 47 mm in zone 0, extending from the aortic root to the iliac arteries (Figures 1A and 1B). The patient underwent emergent surgical repair with right axillary cannulation and clamping of the innominate artery for antegrade cerebral perfusion, a Bentall procedure with a 27-mm bioprosthetic valve, ascending aortic replacement with a 32-mm graft, and AMDS Hybrid Prosthesis implantation with a 55- to 40-mm tapered stent (Figures 1C and 1D). Postoperatively, the patient had an acute kidney injury, which resolved. He was discharged on postoperative day 10.

**Figure 2:**
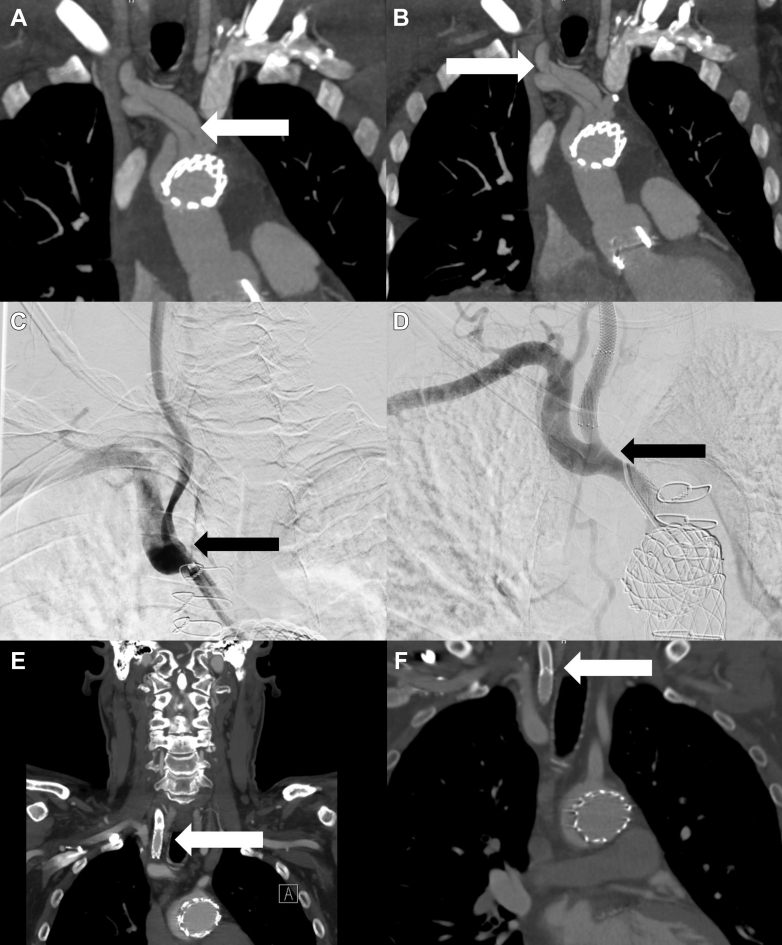
Postoperative Computed Tomography Scans Postoperative computed tomography (CT) demonstrating dissection of the innominate artery (A) and right common carotid artery (B), with re-entry tears present. Intraprocedural imaging from the head vessel stenting procedure demonstrating filling of the innominate artery false lumen prior to stenting (C), with resolution of false lumen filling poststenting (D). Postprocedure CT demonstrating a stent in the innominate artery (E) and right common carotid artery (F). Arrows indicate the area of interest.

A predischarge computed tomography identified a reduction in aortic diameter compared to preoperative measurements in zones 0 to 5, with diameters ranging from 36 mm in zone 2 to 41 mm in zone 4. Follow-up imaging at 1 year revealed a dilating distal aorta with growth of 2 mm in zone 0, 4 mm in zone 1, 9 mm in zone 2, and 7 mm in zone 4. True lumen to false lumen (FL) communications, or tears, were identified postoperatively in the proximal innominate artery (IA) and right common carotid artery (RCCA), just distal to the bifurcation (Figures 2A and 2B).

## Management

The patient was referred for endovascular stenting of his IA and RCCA approximately 1 year after his initial aortic repair. A 10 × 27 mm BeGraft covered stent was placed in the innominate artery, and the RCCA was treated with a 7 × 50 mm Boston Wallstent (Boston Scientific) and an 8 x 30 mm EV 3 Protégé stent (Medtronic) (Figures 2C to 2F). He was discharged on postoperative day 1 without complications.

## Outcomes and Follow-Up

Follow-up imaging was performed annually, revealing stabilization of the aortic arch. Following endovascular intervention, zones 0 to 3 had no further growth up to 3 years post-reintervention. Zones 4 and 5 continued to have gradual growth of the FL due to a tear identified at the base of the left subclavian artery, which continues to perfuse the FL. Given the gradual growth of 4 to 5 mm over 3 years, surveillance is planned for his descending aorta with no indication for intervention at this time. In the event of further adverse remodeling thought to be related to the left subclavian artery, further intervention may be required.

## Discussion

Traditionally, adverse aortic remodeling has been attributed to perfusion of the FL via aortic re-entry tears, distal anastomotic new entry tears, or in cases of stent implantation, distal stent–induced new entry tears. The role of branch vessel involvement has received little attention until recently, with the majority of investigations into this topic being retrospective studies.

Branch vessels have been proposed to contribute to adverse aortic remodeling through dissection and re-entry tears. These re-entry tears are proposed to result in retrograde flow into the FL of the aorta and pressurization of the FL. There have been previous investigations into this topic that have suggested a relationship between branch vessel involvement and aortic degeneration following ATAAD repair. A previous study by Heo and colleagues investigated the impact of intimal tear location and abdominal vessel involvement on aortic remodeling following total arch repair for ATAAD. Their study identified the number of visceral vessels arising from the FL to be associated with total aortic diameter growth.^6^ A substudy of the DARTS (Dissected Aorta Repair Through Stent) trial conducted by our group similarly identified visceral vessel involvement to be associated with distal aortic remodeling and supra-aortic vessel involvement to be associated with proximal aortic remodeling.^2^

While this relationship has been demonstrated, evidence has been limited to retrospective studies and correlations, without causal effects shown. This case report demonstrates a clear relationship between branch vessel involvement and adverse aortic remodeling; in this case, involvement of the RCCA and IA was associated with adverse aortic remodeling and treatment of these branch vessels alone resulted in cessation of aortic growth, demonstrating the association between branch vessel involvement and aortic remodeling. In the case where further intervention of the left subclavian artery is required, endovascular intervention may be pursued, although it would require an adequate landing zone and absence of aortic re-entry tears in the region. If these criteria are not met, surgical intervention of the left subclavian artery, including ligation and bypassing, may be required.

The relationship between branch vessel involvement and aortic remodeling is of considerable importance as it impacts the management of these patients. The ability to treat branch vessels endovascularly resulting in slowing of aortic enlargement would avoid the need for open reoperation of the aorta following ATAAD repair with adverse postoperative remodeling in a subset of cases. In cases of adverse aortic remodeling following ATAAD repair, involvement of the local branch vessels should be considered, as effective treatment of these branches, in the absence of aortic re-entry tears, may resolve adverse aortic remodeling.

## Funding Support and Author Disclosures

Dr Moon has received consulting fees from Artivion. Dr EL-Andari has received support for this work from the Vanier Canada Graduate Scholarship through Canadian Institutes of Health Research (CIHR). Dr Rempel has reported that he has no relationships relevant to the contents of this paper to disclose.

**Take-Home Message**

• Previous investigations have demonstrated an association between adverse branch vessel involvement and adverse aortic remodeling, although a causal relationship has not been conclusively proven.
• This case report demonstrates the cessation of aortic arch degeneration following endovascular treatment of the RCCA and IA.
• Endovascular intervention to treat branch vessel involvement should be considered in cases of adverse aortic remodeling following ATAAD repair, with tears identified in any major branch vessels.
